# Low-cost Solutions for Velocimetry in Rotating and Opaque Fluid Experiments using Ultrasonic Time of Flight

**DOI:** 10.1007/s40799-021-00469-x

**Published:** 2021-06-30

**Authors:** Fabian Burmann, Jerome Noir, Stefan Beetschen, Andrew Jackson

**Affiliations:** Institute of Geophysics, ETH Zurich, Sonneggstrasse 5, 8092 Zurich, Switzerland

**Keywords:** Ultrasonic flow velocimetry, Time of flight, Rotating fluids, Opaque fluids

## Abstract

Many common techniques for flow measurement, such as Particle Image Velocimetry (PIV) or Ultrasonic Doppler Velocimetry (UDV), rely on the presence of reflectors in the fluid. These methods fail to operate when e.g centrifugal or gravitational acceleration leads to a rarefaction of scatterers in the fluid, as for instance in rapidly rotating experiments. In this article we present two low-cost implementations for flow measurement based on the transit time (or Time of Flight) of acoustic waves, that do not require the presence of scatterers in the fluid. We compare our two implementations against UDV in a well controlled experiment with a simple oscillating flow and show we can achieve measurements in the sub-centimeter per second velocity range with an accuracy of $\sim 5-10\%$. We also perform measurements in a rotating experiment with a complex flow structure from which we extract the mean zonal flow, which is in good agreement with theoretical predictions.

## Introduction

The field of rapidly rotating experiments has been growing in the last 30 years, in particular in the context of planetary core and subsurface ocean dynamics. Two major ingredients are necessary to simulate the dynamics of planetary cores, a rapid rotation rate and a high electrical conductivity of the fluid. Due to the opacity of liquid metal, optical methods such as Particle Image Velocimetry (PIV) or Laser Doppler Velocimetry (LDV) fail to operate. As a consequence, Ultrasonic Doppler Velocimetry (UDV) became the most common technique for measuring flow velocities in liquid metal experiments (e.g. [1–3]). UDV works by emitting acoustic bursts into the fluid that are reflected back by seeds suspended therein. The analysis of the back scattered energy yields time-resolved velocity profiles in the direction of the acoustic path.

In the case of rapidly rotating experiments (500 rpm to 10000 rpm, corresponding to centrifugal accelerations of 25g to 10 000g in a typical experiment with 10 cm radius), such as the ones relevant for planetary applications, the reflecting particles will be inevitably centrifuged by the rotation due to the small, yet finite, density contrast between particles and the fluid. After a certain time that depends on the rotation rate and the density contrast, the bulk of the fluid will be depleted in reflectors and UDV measurements are no longer possible.

In such cases, it is still possible to measure flow velocities with transit time or Time of Flight (ToF) methods, which rely on the travel time difference between up- and downstream traveling acoustic waves. From the travel time difference an averaged value for the flow velocity along the acoustic path is calculated.

Since the 1960s, transit time methods have been widely used in medical applications, mostly for intravascular blood flow measurements (see, for example, [4–6]) and to monitor flow rates in pipes (e.g. [7, 8]). With recent progress in the sensitivity and miniaturization of electronic hardware, ToF is an alternative to UDV, LDV and PIV in rapidly rotating experiments, when seeding of the flow with particles is not possible. However, it comes with the drawback, that ToF only provides an integrated value of the velocity over the length of the ultrasonic path and there is no spatial resolution of the flow velocity. Recent studies have also introduced the concept of modal acoustic tomography, that relies on the rotational splitting of acoustic normal modes, to extract flows in the absence of tracer particles [9–11]. This method has successfully been implemented using gas as a fluid medium and bears potential for measurements in other opaque fluids such as liquid metals.

In this study we present two ToF measurement systems based on commercially available parts. One system is based on a lock-in amplifier and is well suited for high precision measurements. The second solution is based on a low-cost (less than 100 CHF) Texas Instruments evaluation board, characterized by light weight and a small footprint ($\sim {10} \text {cm} \times {12}$cm), both of which are quite desirable features in rapidly rotating experiments. In the supplementary material, we provide a detailed description of technical information regarding the instruments and post processing routines, which are also available upon request.

We start by reviewing the ToF principle and its interpretation in terms of a travel time delay and associated phase shift in “The Time of Flight Principle”. Subsequently, we introduce two implementations of ToF measurements in “Methods and Instruments”. We then seek to compare the two measurement systems in a simple uniform flow before we apply them to measure flow velocities in a typical experiment of rotating fluid dynamics. The experimental details of these two test cases are introduced in “Experimental Validation” and the results are presented in “Results”.

## The Time of Flight Principle

Time of Flight velocimetry measures the travel time of an acoustic wave emitted by a transducer *A* and received by a transducer *B*. In the absence of flow the travel time is given by *τ* = *D*/*c*, with *D* the distance between transducers *A* and *B* and *c* the sound speed in the medium. A flow **v** in the fluid will advect the wave, leading to a small perturbation of the travel time *δτ*, as (e.g. following [12]):

1 $$ \begin{array}{@{}rcl@{}} \frac{\delta \tau}{\tau} = -{\int}_{\mathcal{L}} \frac{\textbf{v}\cdot\textbf{k}}{c |k|}dl, \end{array} $$

where **k** is the wave vector of the acoustic wave, *c* denotes the speed of sound, and the integral is taken along the ray path of the acoustic wave. Let us consider the following simple example, where the background flow is of uniform velocity magnitude *v*, and in a direction from *A* to *B*. The advected wave will arrive at receiver *B* after a travel time *τ*_*AB*_ given by

2 $$ \begin{array}{@{}rcl@{}} \tau_{AB} = \frac{D}{c}\left( 1- \frac{v}{c}\right). \end{array} $$

The arrival time at the receiver is not only affected by the flow amplitude *v*, but also by the temperature field in the fluid or the unknown transfer function of the transducers, both of which can lead to a false interpretation of *τ*_*AB*_. To overcome these uncertainties, classical ToF measurements compare upstream and downstream traveling waves by forming a differential travel time (Δ*τ*), to infer the flow velocity using

3 $$ \begin{array}{@{}rcl@{}} v = \frac{\frac{1}{2}(\tau_{BA}-\tau_{AB})c}{\tau} = \frac{\Delta \tau }{2\tau} c. \end{array} $$

Alternatively, the time delay can also be interpreted in terms of a phase shift between the unperturbed and the advected wave. In this framework, the flow velocity is deduced from

4 $$ \begin{array}{@{}rcl@{}} v = \frac{\frac{1}{2}(\varphi_{BA}-\varphi_{AB})c^{2}}{2 \pi f_{e} D} = \frac{c^{2}{\Delta}\varphi}{4\pi f_{e} D} , \end{array} $$

where *φ*_*AB*_ and *φ*_*BA*_ denote downstream and upstream phase shift and *f*_*e*_ is the frequency of the emitted wave. In this article, we use both approaches and their implementations will be explained in the following section.

## Methods and Instruments

### Phase shift detection

In a first approach we measure the phase shift associated with the advection of acoustic waves by demodulation of the received signal.

**Fig. 1:**
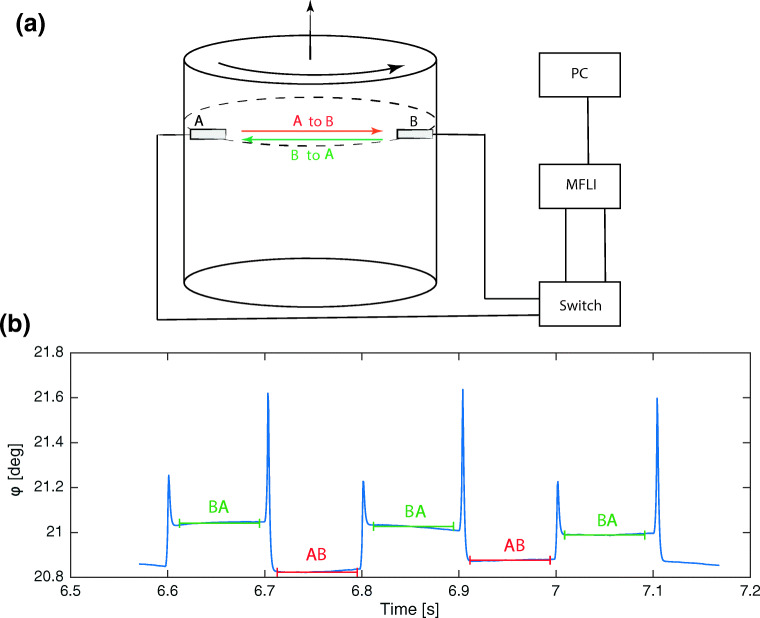
**a** Components of the phase shift detection system. **b** Identification of phase shift from up and downstream traveling waves. Red lines mark averaging of the phase for direction $A \rightarrow B$ and green lines for direction $B \rightarrow A$

The main part of the measurement system is a *MFLI* lock-in amplifier fabricated by *Zürich Instruments*.^1^ A sketch of all components of the system is presented in the upper panel of Fig. 1.^2^ The *MFLI* lock-in amplifier is equipped with a built-in signal generator that is used to feed a 2 MHz continuous sine wave with an amplitude of 100 mV to a transducer. The same sine wave is also used as a reference signal for the demodulation. The transmission of the signal at A and B is done via standard 2 MHz ultrasonic transducers, commercially available at *Signal Processing SA,1073 Savigny, Switzerland*.

To perform differential measurements, i.e. to compare *φ*_*AB*_ and *φ*_*BA*_, we need to exchange emitting and receiving probes. To that end, a home-made switch is used to exchange emitting and receiving probes at a maximum switching frequency of approximately 20 Hz. The switch consists of two mechanical relays, controlled by an *Arduino Uno* micro controller. At the receiver *B*, the incoming signal is digitized by the lock-in amplifier at a frequency of 60 MHz and the phase shift between reference signal and recorded signal is determined by demodulation using the reference signal.

The *MFLI* performs the demodulation and writes the data in complex valued rectangular form as IQ signals, where *I*(*t*) is the in-phase component of the signal and *Q*(*t*) is the quadrature component of the signal. From *I*(*t*) and *Q*(*t*) we calculate the amplitude $A(t) = \left [I(t)^{2}+Q(t)^{2}\right ]^{1/2}$ and phase of the signal by $\varphi (t) = \tan ^{-1}\left [Q(t)/I(t)\right ]$.

We then split the signal into *φ*_*AB*_ and *φ*_*AB*_ and the phase shift of a single switching interval is averaged over a user defined number of samples, equally distributed around the center of the switching period (see bottom panel of Fig. 1). From this, we calculate the differential phase shift Δ*φ* = *φ*_*AB*_ − *φ*_*BA*_, which we use to evaluate the flow velocity using Eq. 4 at a maximum sampling frequency of 10 Hz. A higher sampling frequency can be achieved by increasing the switching frequency between $A \rightarrow B$ and $B \rightarrow A$, at the cost of a higher noise level. The maximum resolvable velocity without aliasing *v*_*max*_, i.e. when the phase shift corresponds to a full cycle of the wave is given by:

5 $$ \begin{array}{@{}rcl@{}} v_{max} = \frac{ 2 c^{2}}{f_{e} D}. \end{array} $$

While *c* and *D* are parameters given by the setup of the experiment, *f*_*e*_ will be chosen according to the maximum expected velocity and the peak of resonance of the piezo-electric sensor in the transducer.

### Detection via Time Delay

The flow velocity can be directly determined from the time delay introduced by the advection of the acoustic wave via Eq. 1. The measurement system is based on a standard, commercially available TDC1000^3^ ultrasonic analog-front-end (AFE) connected to a TDC7200EVM evaluation module. The evaluation module makes use of the TDC7200 time-to-digital converter and is commercially available from *Texas Instruments*. We connect the evaluation board to two standard 2 MHz UDV transducers produced by *Signal Processing SA*. The evaluation board is controlled via a graphical user interface (GUI) written in PySide2 (Qt for Python).^4^

The first transducer (emitter *A*) is used to emit an ultrasonic burst into the fluid. After the burst has traveled through the fluid, it is recorded at the second transducer (receiver *B*). The TDC1000 detects the rising edge zero-crossing of the oscillations after exceeding a user defined threshold voltage, and up to five edges can be detected. The internal reference clock of the evaluation board is used to measure the time delay between the emission and the detected rising edge. Our software writes the time stamp of each detected rising edge *τ*_*i*_ for an emitted burst in units of ns. The maximum sampling frequency of the system is up to 10 Hz.

We note, that *τ*_*BA*_ and *τ*_*AB*_ for the *i*^th^ rising edge are not exactly equal in the absence of flow, which can be accounted for by a calibration of the differential travel time *τ*_*BA*_ − *τ*_*AB*_ in the absence of flow.^5^ We take the average over the five rising edges and apply Eq. 3 to translate the differential travel time into the flow velocity *v*.

## Experimental Validation

### Experimental Apparatus

**Fig. 2:**
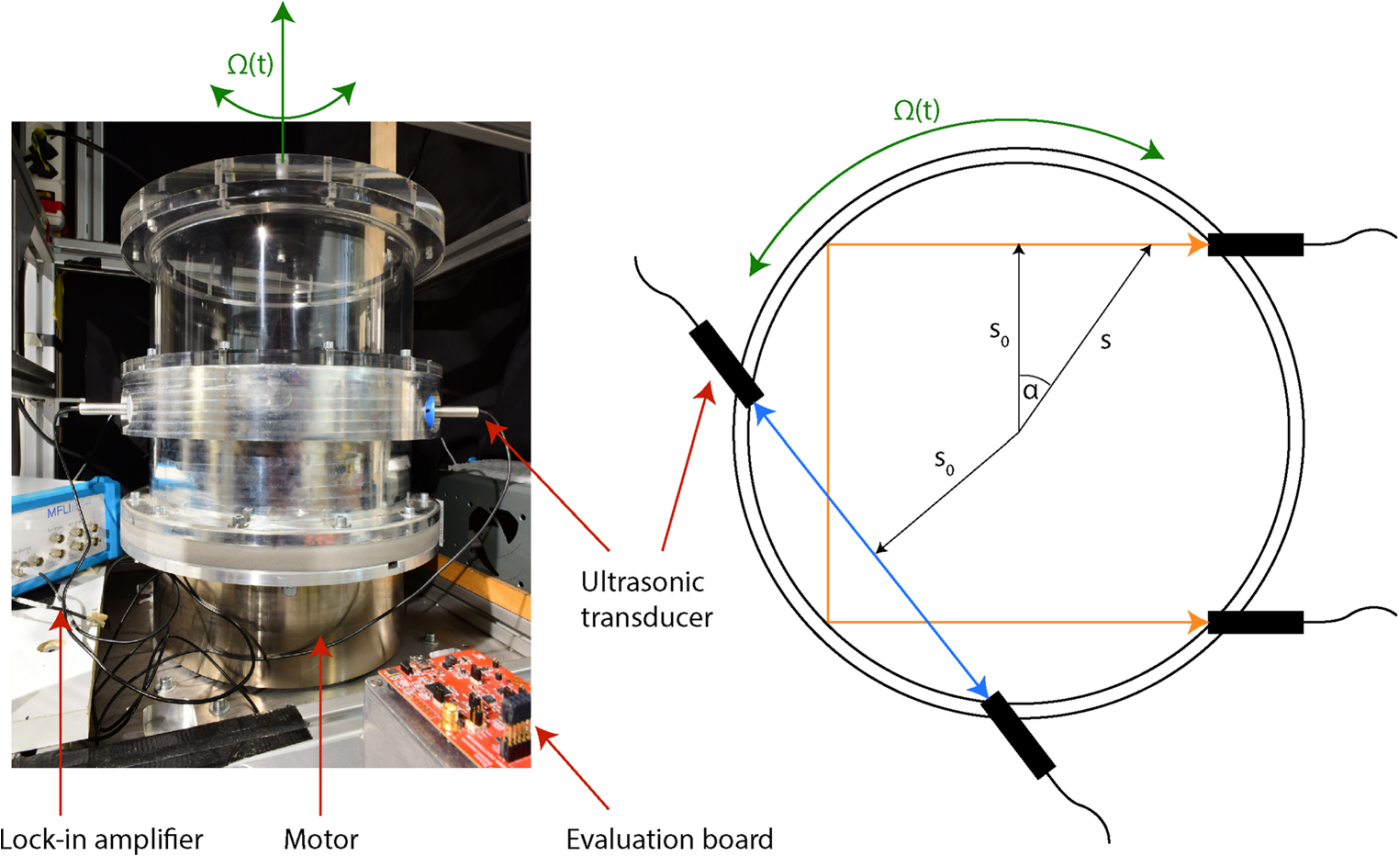
Photograph of the apparatus for the oscillating flow experiments. The entire device is sitting on a turntable that allows us to impose a global rotation. The sketch on the right displays the geometry of the transducer placement, showing *s*_0_ to be the distance of closest approach of the acoustic ray to the centre of the cylinder

To validate our two measurement systems, we perform experimental measurements in an oscillating, straight cylinder filled with water. An illustration of the apparatus is presented in Fig. 2 and the relevant experimental parameters are given in Table 1.

**Table 1:** Definition of physical parameters and the corresponding values in the experimental validation of the two ToF-systems

Parameter	Definition	Experiment
*R*	Radius of the cylinder	14.2 cm
*H*	Height of the cylinder	28.6 cm
*s*_0_	Cylindrical radius perpendicular to the ray	9.9 cm
*f*_*e*_	Frequency of the ultrasonic wave	2 MHz
*D*_*d*_	Length of the ray path (direct)	19.5 cm
*D*_*r*_	Length of the ray path (reflected)	58.9 cm
*c*	Sound speed at 22^∘^*C*	1480 m s^− 1^
*ν*	Kinematic viscosity of water	1× 10^− 6^ m^2^ s^− 1^
*f*_0_	Imposed oscillation frequency of the tank	0.1 Hz to 0.7 Hz
ΔΦ	Imposed oscillation angle	0.03 rad to 0.26 rad
*A*_0_	Imposed amplitude of the oscillations	0.02 s^− 1^ to 0.1 s^− 1^
*f*_*U*_	Sampling frequency of the UDV	10.3 Hz
*f*_*φ*_	Sampling frequency of the phase shift detection	5 Hz to 10 Hz
*f*_*τ*_	Sampling frequency of the travel time detection	10 Hz
*f*_*M*_	Sampling frequency of the motor logger	100 Hz

The cylinder (14.2 cm in radius *R* and 28.6 cm in height *H*) is mounted on a Yakasawa SGMCS-35E5B11 direct drive that is controlled by a SGDV-5R5A11A servopack connected to a personal computer, accessed remotely via wifi. We use this motor to apply time harmonic oscillations of the container. The entire experiment (container and motor) is mounted on a turntable with the same specifications, which allows us to impose a global rotation Ω_0_. We record torque, speed and position of the motor and the respective errors at a sampling frequency of 100 Hz.

We use four transducers, two forming a direct ray path and the other two forming a ray path with two reflections, as illustrated in Fig. 2. All transducers are in the same plane 7 cm above the bottom of the container. We use standard 2 MHz ultrasonic transducers, commercially available at *Signal Processing SA,1073 Savigny, Switzerland*. The ultrasonic transducers are attached to the cylinder through holes in the side wall and are in direct contact with the fluid. The cylinder is moving at

6 $$ \begin{array}{@{}rcl@{}} {\Omega} (t) = {\Omega}_{0} + A_{0} \sin(2 \pi f_{0} t), \end{array} $$

where Ω(*t*) is the instantaneous rotation rate of the container, Ω_0_ is the global rotation, and *A*_0_ = 2*πf*_0_ΔΦ denotes the amplitude of the oscillations depending on the oscillation angle ΔΦ in radians and *f*_0_ the oscillation frequency in Hz.

### Reference measurements with UDV

In the present experiment we aim at evaluating the performance of our travel time detection device and the lock-in amplifier solution and compare it to a UDV instrument (DOP3010 produced by *Signal Processing SA,1073 Savigny, Switzerland*). In contrast to the ToF measurements, the UDV technique gives time-resolved velocity profiles along the ray path.

We use the UDV technique in a similar way to the ToF-systems, by averaging the recorded velocities over the length of the UDV chord in the post-processing. To exclude the effects of acoustic streaming [13] and effects of transducer saturation, we generally exclude all data points located closer than 5 mm to the transducer from our averaging. Furthermore, we carefully monitor the data quality and exclude data points from the average whenever the data quality in some parts of the UDV chord is too low, an effect that is almost inevitable at very low velocities.

The DOP3010 is connected to the same 2 MHz transducers as in our ToF measurements. The fluid is seeded with a mixture of 2AP1 Particles produced by *Griltex* of sizes 50 mm (60% by weight) and 80 mm (40% by weight) with a density of 1.02 g cm^− 3^.

### Experimental Protocol

The experimental protocol for all experiments is the following: we leave the fluid in the tank at rest for about 30 s, before we start the ToF measuring systems. We then start recording torque, speed and position of the motor. After another 20 s, we set the cylinder into motion at Ω(*t*) and record with the ToF systems for about 2–3 minutes. We then repeat the experiment at a different amplitude and frequency of the oscillation, following the same procedure. For both the motor and the measurement time series, we visually identify the starting time of the oscillations *t*_0_.

## Results

### In the Absence of Flow

**Fig. 3:**
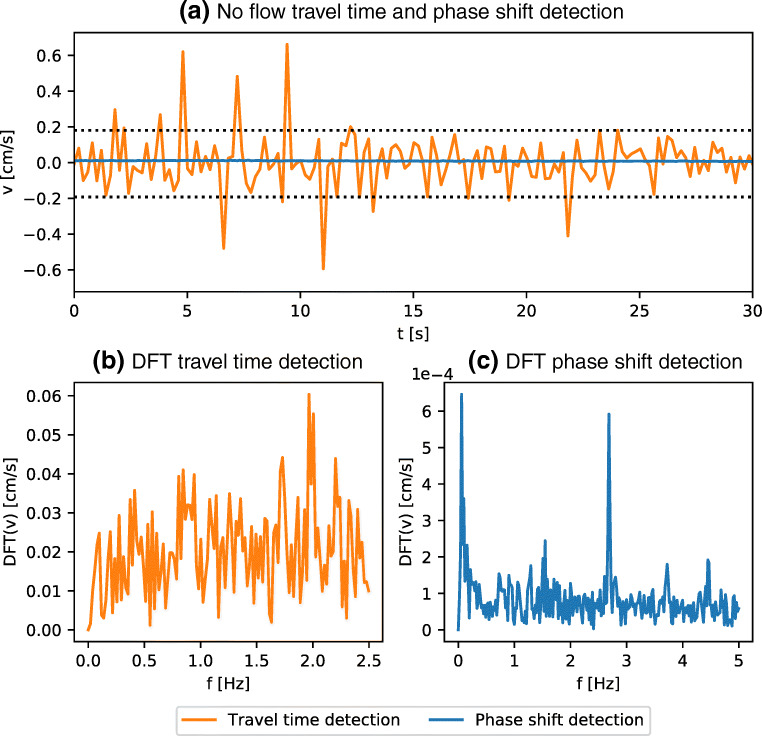
Examples of the recorded velocities in the absence of background flow. **a** In orange for time measurements and blue for phase measurements. The dotted lines are the 5th and the 95th percentile of the measured velocities with the travel time detection. **b** and **c** show the amplitude of the Discrete Fourier Transform (DFT) of the two time series. Note the different scales in **b** and **c**

The variation of recorded velocities in the absence of a flow field gives a first idea of the noise level for both measurement systems. Figure 3a displays typical time series while the tank is at rest, for both the travel time detection system and the phase shift detection system. Both time series are representative of all other conducted experiments. We use the 5th and the 95th percentile of the measured velocities (dotted lines in Fig. 3a to define a typical amplitude of the noise floor for both systems. It is expected that the phase shift detection results in a much lower noise floor at $\sim 2\times 10^{-3} \mathrm {cm~s}^{-1}$ than the travel time detection, which exhibits fluctuations on the order of $\sim {0.2}$ cm s^− 1^ for the present configuration. Looking at the Discrete Fourier Transform (DFT) of the two time series, the travel time detection exhibits a broad noise spectrum Fig. 3b, while the phase shift detection is characterized by two peaks, as shown in Fig. 3c.

In addition to the rapid oscillation of the velocity, we observe a long period background drift, which corresponds to the peak at low frequency of the spectrum displayed in Fig. 3c. The amplitude of the drift remains small but would put a limit on the lowest velocities that can be measured over a very long period of time. We could not identify the origin of this drift, present only in the phase shift detection. The main difference between the two systems, aside from the detection method, resides in the excitation. While the TI evaluation board, used in the travel time detection, sends bursts, the lock-in amplifier uses continuous emission. Using an independent wave generator and an oscilloscope, we performed ToF measurements using continuous wave excitation and observed the same low frequency drift, from which we conclude it is not inherent to the MFLI instrument.

### Uniform Oscillating Flow

First, we aim to validate our measurement systems against the most simple case of a uniform oscillating flow without global rotation (Ω_0_ = 0 in Eq. 6). For a harmonic oscillating boundary in the absence of a global rotation, the transfer of momentum from the boundary to the fluid happens by diffusion in a thin Stokes boundary layer. The penetration depth of the Stokes boundary layer depends on the oscillation frequency *f*_0_ as $\delta _{SL} \sim \sqrt {\nu / 2 \pi f_{0}}$, where *ν* denotes the kinematic viscosity. For our experiments *δ*_*SL*_ is typically on the order of 1 mm. Thus, we can safely assume that the interior of the fluid will not be affected by the oscillations of the walls and that the fluid outside the boundary layer stays at rest in the frame of inertia.

Yet, as seen from the frame of the container and the attached ultrasonic transducers, the fluid performs a solid body oscillation in the form **v**(*r*) = −**Ω**(*t*) ×**r**, with $\boldsymbol {\Omega }(t) = \boldsymbol {\Omega }(t) \hat {e}_{z}$. The cylindrical radius of each point along the ray path can be expressed by $s = s_{0} / \cos \limits \alpha $ (see *s*_0_ in Fig. 2 and Table 1). Hence, the velocity projection along the ray path is constant and equal to

7 $$ \begin{array}{@{}rcl@{}} v(t) = -A_{0} \sin(2 \pi f_{0} t) s_{0}. \end{array} $$

where the product of *A*_0_ and *s*_0_ is representative of the amplitude of the oscillations in cm s^− 1^, i.e. the maximum flow velocity as seen from the viewpoint of the transducers.

**Fig. 4:**
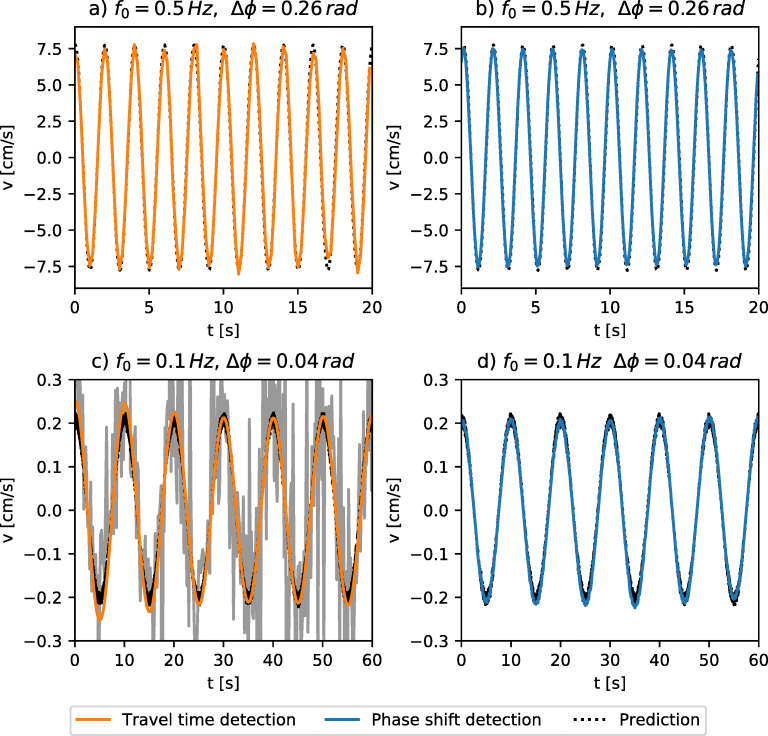
Measurements for large amplitude (**a,b**) and small amplitude (**c,d**) of the apparent velocity in the oscillation flow. Results for the travel time detection are displayed in on the left (**a,c**) and phase shift detection on the right (**b,d**). Together with the experimental data we display the predicted time series following Eq. 7. In figure c) the original measurements are displayed in grey color and the orange time series represents the same time series after applying a narrow band pass filter. Exact experimental conditions are given in the individual panels

We start with a series of experiments using a direct ray path, represented by the blue arrow in Fig. 2. Time series of the velocities for a purely oscillating cylinder (Ω_0_ = 0) at two different values of *f*_0_ and ΔΦ are presented in Fig. 4. For comparison, we additionally display the time series of the expected velocity (Eq. 7 with values for ${\Omega }(t)=-A_{0} \sin \limits (2 \pi f_{0} t)$ obtained from the motor optical encoder) as a dashed black line. For a large enough amplitude of the oscillation, leading to velocities on the order of $\sim {10}$ cm s^− 1^, we see that the phase shift and travel time detection compare equally well with the predictions.

At lower amplitude of the oscillations approaching the noise floor of the travel time detection, we observe significant fluctuations around the predicted velocities (Fig. 4c). Additional filtering of the signal at the forcing frequency *f*_0_ allows us to recover a harmonic signal in agreement with the predicted velocity. The results for the phase shift detection in the same range of small velocities are presented in Fig. 4d), showing very low noise as anticipated from the previous measurements without flow.

To further assess the range of validity of our two ToF systems, we carry out a systematic study for various frequencies *f*_0_ and amplitude ΔΦ. As a quantitative measure, we fit a sinusoidal curve of the form $A\sin \limits (2\pi f t + \varphi )$ to the data of each experiment. We fit the sinusoid using the python-function scipy.optimize.curve_fit.^6^ We use *A*_0_ as an initial guess for *A*, *f*_0_ for *f*, and since we do not know the phase of the signal we take a value of zero as an initial guess. From the resulting fit, we use the amplitude *A* to define the normalized measurement error *𝜖* as:

8 $$ \begin{array}{@{}rcl@{}} \epsilon = \left|\frac{A-A_{0}}{A_{0}}\right|. \end{array} $$

**Fig. 5:**
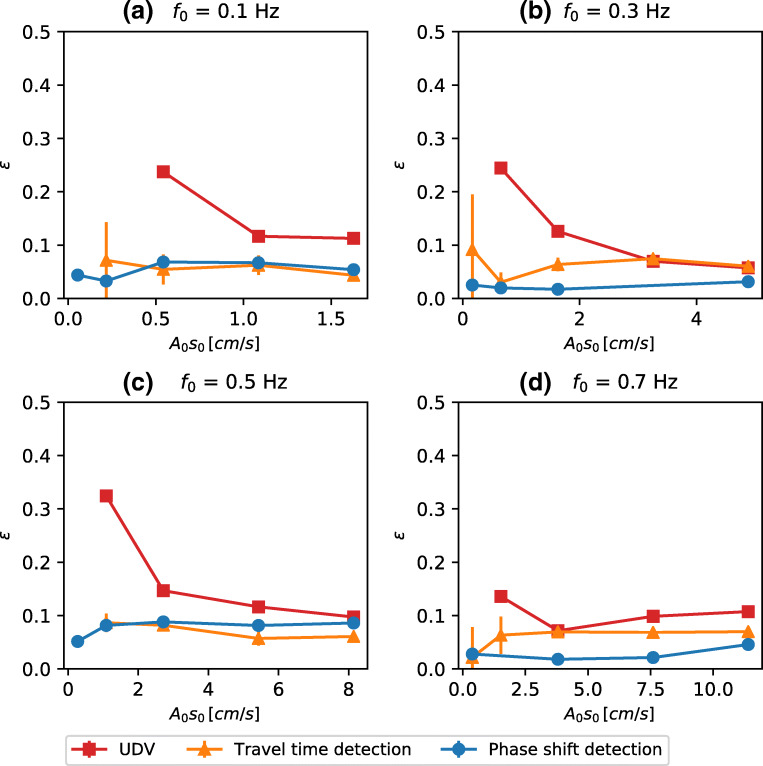
Estimated error in recovering the amplitude of the oscillating flow for all three measurement systems. Different panels represent the different values of of the oscillation frequency

In Fig. 5, we present the normalized error *𝜖* for phase shift detection, travel time detection and UDV for four different frequencies and amplitudes up to 15 cm s^− 1^. The error bars are representative of the standard deviation of the parameter estimate.

While the normalized error for the UDV data decreases rapidly with increasing flow velocity, especially at low frequency as displayed in Fig. 5b-d, both ToF systems show a typical error of < 10*%*, essentially uniform over a wide range of flow amplitudes (0.1 cm s^− 1^ to 15 cm s^− 1^). For all oscillation frequencies, represented by the individual panels of Fig. 5, the ToF methods perform equally well or better than UDV. Moreover, at moderate velocities of 1 cm s^− 1^ to 10 cm s^− 1^, the normalized measurement error is always smaller than 10% and decreases towards values of 5% in Fig. 5c and d.

**Fig. 6:**
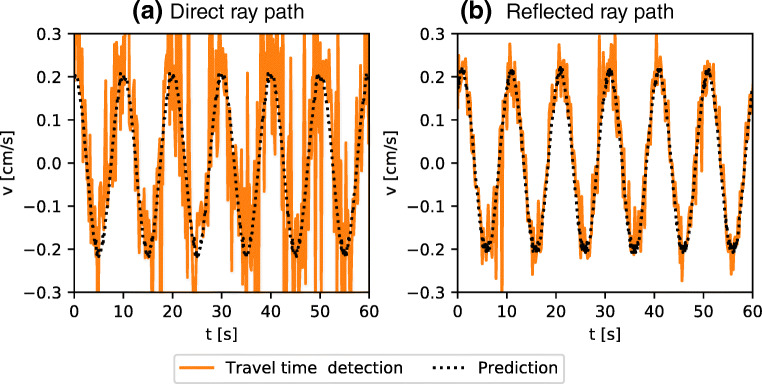
Comparison of the noise level in travel time detection for different length of the ray path. The two time series represent the same experimental conditions in *f* = 0.1 Hz and ΔΦ = 0.04 radians. The time series in **a**) is recorded in the direct ray path configuration and **b**) in the reflected ray path configuration. The prediction is taken from the motor

Anticipating more advanced applications, where the emitted acoustic wave will be reflected multiple times on the side wall of the experiment before it reaches a receiver, we perform a series of measurements using the pair of transducers forming the orange arrow in Fig. 2, for which the emitted wave undergoes two reflections on the side walls. As a general outcome, we observe a significant improvement of the measurement quality. To illustrate this, we show in Fig. 6 a comparison of a velocity measurement with travel time detection without (Fig. 6a) and with (Fig. 6b) reflections along the ray path. While reflections may locally affect the acoustic wave, the main effect of multiple reflections is to increase the total length of the path, leading to a larger Δ*τ* in Eq. 3, hence leading to a larger signal to noise ratio.

### Longitudinal librations

So far, we have set Ω_0_ = 0, leading to a simple solid body rotation oscillating flow in the interior as viewed from the frame of reference attached to the container. In a second experiment, we apply our measurement systems to a typical flow relevant in the field of rotating fluid dynamics. Adding a global rotation (Ω_0_≠ 0 in Eq. 6) results in more complex dynamics, including the excitation of inertial waves and inertial modes. In addition it has been shown [14–16], that non-linear interactions in the top and bottom boundary layers generate a mean zonal flow of the form [17]:

9 $$ \begin{array}{@{}rcl@{}} v_{g}(s) = \left( {\Delta} {\Phi} 2\pi f_{0}\right)^{2} s {\Omega}_{2} \left[1 - \exp\left( -\frac{1-s}{E^{1/4}}\right)\right] \end{array} $$

where Ω_2_ is uniform in the bulk of the fluid and can be calculated from equation A1 in the appendix of [17], and *s* denotes the cylindrical radius. Furthermore $E=\nu /(H^{2}{\Omega }_{0})$ denotes the classical Ekman number of rotating fluid dynamics, measuring the relative importance of viscous forces with respect to Coriolis forces. Hence, the velocity as seen from the frame of reference attached to the container is:

10 $$ \begin{array}{@{}rcl@{}} \textbf{v}(t) = -A_{0}\sin(2\pi f_{0})\hat{e}_{z} \times \textbf{r} + \textbf{v}_{IW} + {v}_{g}\hat{e}_{\phi}, \end{array} $$

where **v**_*IW*_ denotes the contribution of inertial waves and inertial modes. In our experiments we choose the libration frequency *f*_0_ in such a way that there is no direct resonance with the inertial modes in the container, and thus the contribution of **v**_*IW*_ remains small with respect to the oscillations of the cylinder.

We set the table into rotation at 0.5 Hz and later impose harmonic oscillations with *f*_0_ = 0.611 Hz and ΔΦ = 0.235 rad, and wait until a steady state is achieved. Following [15], this is the case after $\sim {157}$ s at the Ekman number in our experiment (4× 10^− 6^).

**Fig. 7:**
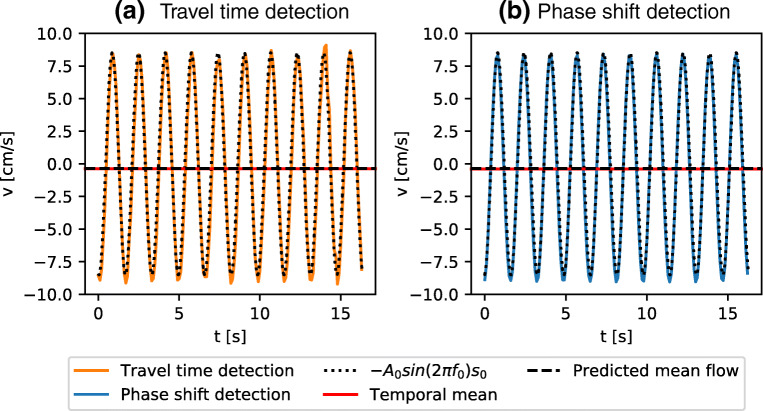
Longitudinal librations as measured from the librating frame recorded with travel time detection (a) and phase shift detection (b). Both time series are recorded in steady state and the black dotted line represents the apparent motion from the oscillations of the container

In Fig. 7 we display time series of the flow velocity measured with travel time (Fig. 7a) and phase shift detection (Fig. 7b). Additionally we display the amplitude of the oscillations that stems from the time harmonic oscillations of the container ($-A_{0}\sin \limits (2\pi f_{0}) s_{0}$). Due to the complex spatial structure of the inertial waves and modes, the contribution of **v**_*IW*_ is difficult to reconstruct from our measurements but should be generally small according to the choice of our experimental parameters. In contrast, the ToF measurement technique is well suited to extracting the mean zonal velocity **v**_*g*_, which we predict from integration of Eq. 9 along the direct ray path (blue arrow in Fig. 2) and which should be retrograde and of amplitude 0.36 cm s^− 1^. Since it is independent in time, we extract the zonal flow by averaging in time over 200 periods of the oscillations displayed in Fig. 7, resulting in -0.396 cm s^− 1^ for the phase shift detection and -0.386 cm s^− 1^ for the travel time detection. Hence, for both system we observe a good agreement of the zonal flow amplitude with our measurements, within 5.4% for travel time detection and within 7.4% for the phase shift detection. This is remarkable as the expected amplitude of the mean flow ($\sim {-0.36 }$ cm s^− 1^) is close to the lower limit of resolvable velocities of the travel time detection of $\sim {0.2}$ cm s^− 1^ as discussed in the section: “In the Absence of Flow”.

## Discussion and Conclusions

In this article we present two solutions for flow velocimetry using the ToF principle. The phase shift detection is based on a lock-in amplifier that performs a digital demodulation of the received ultrasonic wave to determine the phase shift associated with the advection of the acoustic wave by the velocity field. The travel time detection is based on a commercially available Texas Instruments evaluation board that measures the time delay between the emitted and received ultrasonic wave.

By comparing these two systems against a UDV instrument, we characterize the domain of validity of our two measurement systems. We show that, for a typical length of the ray path on the order of 10 cm, we can resolve velocities as low as 0.2 cm s^− 1^ using travel time detection and 0.03 cm s^− 1^ using phase shift detection. Furthermore, we show that reflections of the acoustic waves on the side walls of the container do not degrade the measurements. In fact, the increased length of the ray path leads to an improved signal to noise ratio, hence a better sensitivity towards low flow velocities.

In contrast to UDV or PIV, for which the velocity field is obtained at discrete locations in space, the ToF measurements inherently provide averaged quantities, and thus do not suffer from integration over a discretized domain. Therefore, our two systems are particularly well suited for recovering integrated quantities, such as mean zonal flows, as we demonstrated in the case of longitudinal librations of a straight cylinder.

For experiments where seeding the fluid is impossible, e.g. when suspended particles disappear from the bulk over the duration of the measurements, as for rapidly rotating investigations or when one must use a pure fluid, as in the pharmaceutical or food industry, the ToF method provides a viable alternative to the UDV or PIV technique. Furthermore, the implementation is lightweight, with a small footprint, that can easily be installed on rapidly rotating devices, eliminating the need for slip-rings to transfer signals from the transducer to the acquisition device.

The low-cost solution from Texas Instruments might be used as a building block for a multi-channel simultaneous measurement system, which can be used to overcome the absence of spatial resolution by using an array of transducers forming a ray pattern with multiple intersections. Along this line of thought, [18] have been investigating an adaption of the ToF based on wide band signals and beam forming methods to overcome the limited spatial resolution.

## Footnotes

1 https://www.zhinst.com/ch/en/products/mfli-lock-amplifier

2 For additional technical information about instruments and processing routines we refer the reader to the supplementary information.

3 https://www.ti.com/product/TDC1000-Q1

4 See also the supplementary material for technical information and a detailed description of processing routines.

5 See also the supplementary information for further details.

6 https://docs.scipy.org/doc/scipy/reference/generated/scipy.optimize.curve_fit.html
